# Clinical and Etiopathological Perspective of Vitamin B1 Hypersensitivity and an Example of a Desensitization Protocol

**DOI:** 10.3390/life16010050

**Published:** 2025-12-28

**Authors:** Kinga Lis

**Affiliations:** Department of Allergology, Clinical Immunology and Internal Medicine, Ludwik Rydygier Collegium Medicum in Bydgoszcz, Nicolaus Copernicus University in Torun, ul. Ujejskiego 75, 85-168 Bydgoszcz, Poland; kinga.lis@cm.umk.pl

**Keywords:** vitamin B1, hypersensitivity, desensitization

## Abstract

Vitamin B1 (thiamine) is a water-soluble B vitamin. As a cofactor of many enzymes, it is essential for the proper functioning of many body systems and organs, including metabolic and energy metabolism. In extreme cases, vitamin B1 deficiency causes neurodegenerative disorders, including beri-beri, or cognitive impairment resulting from encephalopathy. B1 avitaminosis may result from increased demand, dietary errors, malabsorption, or excessive loss. Thiamine supplementation is used in cases of vitamin B1 deficiency or for preventative measures in situations of increased demand. Vitamin B1 can be administered enterally or parenterally (intravenously, intramuscularly, subcutaneously). The route and dose depend on the individual patient’s clinical situation. Hypersensitivity to vitamin B1 is rare and appears to be primarily associated with rapid intravenous infusion of large doses of thiamine hydrochloride over a short period (intravenous bolus). Hypersensitivity to thiamine administered by routes other than intravenous or intramuscular injection appears to be an incidental phenomenon. Thiamine should also be considered as an occupational allergen. The mechanism of thiamine hypersensitivity has not been clearly elucidated. However, considering the clinical nature and dynamics of the reaction, the most likely reaction seems to be an immediate type of hypersensitivity reaction (immunoglobulin E (IgE)-dependent), in which thiamine (but not its metabolites) acts as a hapten. Diagnosing hypersensitivity to vitamin B1 is difficult due to the lack of validated tests for additional testing. In individuals requiring thiamine supplementation who have experienced hypersensitivity to intramuscular or intravenous administration of this vitamin, switching to oral administration may be considered (provided this does not reduce treatment efficacy). This form of supplementation is usually well tolerated by individuals allergic to parenteral thiamine. However, if enteral supplementation does not guarantee the maintenance of therapeutic potential, thiamine desensitization may be considered, which seems to be an effective therapeutic method in such a clinical situation.

## 1. Introduction

Vitamins are a group of organic chemical compounds with diverse structures that are essential for the proper functioning of living organisms. All vitamins are exogenous substances and, as such, are not produced in the human body. An exception is vitamin D, which is not a classic vitamin, but a steroid hormone. Small amounts of some B vitamins can be synthesized by endogenous intestinal flora, but the amounts produced in this way are insufficient to meet the human body’s needs for this group of compounds. Therefore, vitamins must be supplied externally, usually through food or supplementation via various routes (e.g., enteral, intramuscular, intravenous). They can be supplied in the form of provitamins or active substances. Vitamins can be derived from natural sources (foods, primarily plant-based, and extracts from natural sources) or obtained through chemical synthesis. Both synthetic and natural vitamins can be components of pharmaceutical preparations, dietary supplements, cosmetics, and food additives (so-called fortified foods) [1,2,3].

Vitamin B1 (thiamine), the other B vitamins, and vitamin C form a common group of water-soluble vitamins. This group of vitamins is widely present in many foods, especially vegetables and fruits, as well as dairy products, meat, legumes, peas, liver, eggs, and enriched grains and cereals. They primarily act as cofactors in biochemical reactions crucial for the proper functioning of the body. B vitamins are essential for, among other things, proper growth and development, the proper functioning of the nervous system, skin, heart, and circulatory system. They also play a significant role in blood formation and modulate the immune system [4,5,6].

Vitamin B1, like other water-soluble vitamins, is very unstable and easily degraded [7]. Water-soluble vitamins are easily absorbed from the intestines, rapidly penetrate tissues, are excreted by the kidneys, and, in most cases, do not accumulate in the body [8,9].

Vitamins, especially those derived from natural sources, are perceived as safe substances that do not cause adverse reactions, including allergic reactions. However, the few published clinical case reports suggest that these substances may be a rare trigger for hypersensitivity reactions with varying symptoms and severity. Because the literature describes few clinical events that clearly link a hypersensitivity reaction to exposure to specific vitamins, it is possible that these events are very rare or that their incidence is underestimated.

## 2. Purpose and Method of the Review

This narrative review focuses on the analysis of published data on vitamin B1 hypersensitivity. Reported clinical cases, in which vitamin B1 was confirmed as the causative agent or thiamine was identified as the most likely factor, were analyzed. Particular attention was paid to the clinical symptoms of vitamin B1 hypersensitivity reactions, potential etiological mechanisms, and current diagnostic and therapeutic options for thiamine hypersensitivity.

Data were searched using keywords in PubMed, Google Scholar, and publicly available internet search engines (e.g., Google). Examples of search terms included vitamin B1 hypersensitivity, vitamin B1 allergy, thiamine hypersensitivity, thiamine allergy, etc. Both English and non-English literature was analyzed.

## 3. Vitamin B1 (Thiamine)–History of Discovery and Basic Information

Vitamin B1 (thiamine, aneurine) belongs to a broad group of vitamins known as the B vitamins (thiamine, riboflavin, niacin, pantothenic acid, pyridoxine, biotin, folic acid, and cobalamin) [2,3,10,11,12,13]. Thiamine was the first recognized substance from a group of organic chemical compounds with a diverse structure that are essential for the proper functioning of living organisms, collectively known as vitamins [14]. Because vitamin B1 is both an amine and essential for life, the combination of these two characteristics gave rise to the name “vitamin” (literally “amine essential for life”) [15]. The name “vitamin” was proposed in 1913 by the Polish biochemist Kazimierz Funk, after he characterized and described thiamine. Although it is currently known that while all vitamins are necessary for the proper growth, development and functioning of the body, not all of them are amines, the term “vitamin” still describes a group of exogenous substances that are necessary for the proper development and functioning of the body [14,15].

### 3.1. History of Discovery of Vitamin B1

The history of the discovery of thiamine is inextricably linked to the history of research on beri-beri, which dates back to the 1880s [16]. Beri-beri (known as “kakké” in Japan and China) is a disease characterized by impaired function of the nervous and circulatory systems caused by extreme vitamin B1 deficiency. The name “beri-beri” was likely borrowed in the 18th century from the Sinhalese phrase “bæri bæri,” meaning “I can’t, I can’t,” and refers to the neuromuscular weakness that is the main symptom of the disease. The first descriptions of a set of characteristic symptoms caused by vitamin B1 deficiency appeared in the first half of the 18th century, and were initially suspected of being infectious [14,16].

In the 19th century, Dutch physician and researcher Christiaan Eijkman noticed that chickens fed highly refined rice developed symptoms of beri-beri, and that adding rice husks to the birds’ diet reversed this effect. Initially, he suspected that white rice contained toxins that caused damage to the nervous system, and that rice husks were a source of an antidote that neutralized these toxins. In 1929, Eijkman was awarded the Nobel Prize in Physiology or Medicine for his work on beri-beri [16,17]. Research conducted by K. Funk showed that Eijkman’s conclusions were not entirely accurate. Funk demonstrated that white rice does not contain neurotoxins, but rice bran contains a substance whose deficiency causes polyneuritis in birds and beri-beri, and that this substance is not present in white rice [14,16]. In 1912, Funk isolated and characterized this substance and named it vitamin [18,19]. The molecular and structural formula of vitamin B1 was determined by Robert Williams, who synthesized thiamine for the first time in 1936 [14,16].

### 3.2. Physical and Chemical Properties, Natural Sources and Metabolism of Vitamin B1

**Figure 1:**
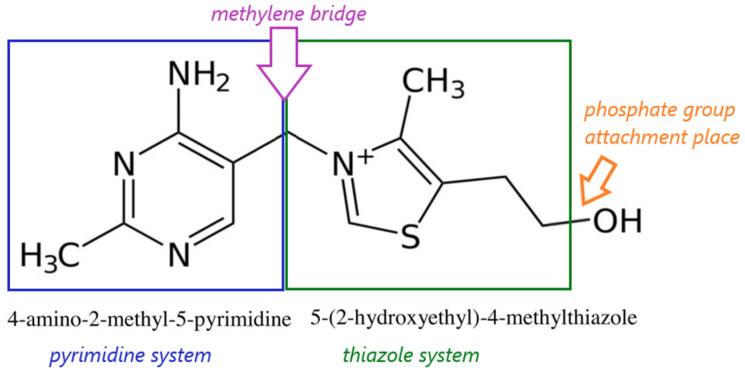
Structural formula of thiamine; author’s own figure based on [20,21,22,23].

Thiamine is a pyrimidine and thiazole derivative. The thiamine molecule consists of two heterocyclic rings, forming a pyrimidine and thiazole system, connected by a short methylene bridge (Figure 1) [20,21,22,23].

**Figure 2:**
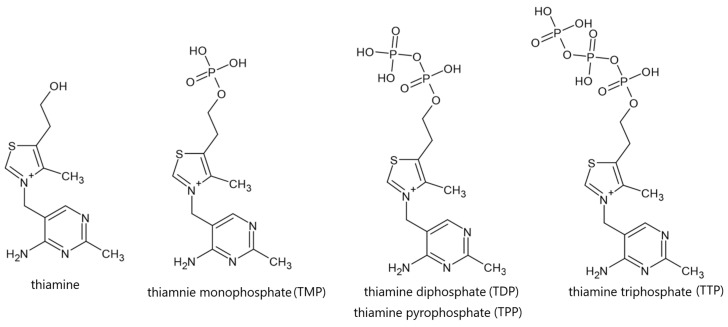
Thiamine and thiamine phosphates; author’s own drawing based on [20,22,24].

Thiamine occurs in free form or in the form of phosphoric esters, as thiamine monophosphate, diphosphate or triphosphate (Figure 2). Individual chemical forms of vitamin B1 have different biological and physical properties, including metabolic activity and the ability to interact with the immune system, including the potential to induce an immune response [20,21,22,23,24].

Thiamine phosphate esters are biologically active forms of vitamin B1 that perform key metabolic functions. Thiamine diphosphate (thiamine pyrophosphate, cocarboxylase) is the most important metabolically active form of thiamine. It is a coenzyme essential for many enzymes (including pyruvate dehydrogenase, alpha-ketoglutarate dehydrogenase, and transketolase) involved in key metabolic pathways, including carbohydrate and amino acid metabolism and the release of energy from carbohydrates through the breakdown of pyruvate to acetyl-CoA. Thiamine pyrophosphate is essential for key reactions in the nervous system and energy metabolism, as well as for the synthesis of ribonucleic acid (RNA), deoxyribonucleic acid (DNA), and neurotransmitters (e.g., acetylcholine (Ach)). It also participates in the formation of myelin sheaths and the maintenance of normal levels of glutamate, aspartate, and gamma-aminobutyric acid (GABA) in the central nervous system. Vitamin B1 deficiency increases the concentration of pyruvic acid in tissues, leading to tissue damage. In the nervous system, ACh and GABA concentrations decrease, while glutamate concentrations increase, leading to neuronal excitation and impaired consciousness [20,21,22,23,24,25,26]. Thiamine likely also serves functions other than coenzymatic functions in the body, but the role of vitamin B1 in this area is still poorly understood. High concentrations of thiamine triphosphate are found in erythrocytes, skeletal muscles, and nerve cells, where it likely participates in energy processes and adenosine triphosphate (ATP) synthesis [20,27].

The main source of vitamin B1 is a balanced diet. Foods rich in thiamine include mainly legumes, nuts, whole grains, cereals, cereal sprouts, seeds, pork, poultry, eggs, fish, and yeast (Table 1). Thiamine in foods of animal origin occurs mainly in phosphorylated forms, while in plant products the non-phosphorylated form predominates [20,21,24,28]. Refined or processed cereal products (e.g., polished rice, refined flour) contain small amounts of vitamin B1 or are completely devoid of it [24,29,30]. Processed foods (e.g., cereals, bread, dairy products) or foods for special nutritional uses (e.g., nutritional supplements, infant formula) are fortified with thiamine to compensate for losses resulting from technological processes [31,32].

Thiamine is well absorbed in the jejunum and ileum through an active, carrier-dependent, and rate-limited process. In high dietary intakes, absorption likely occurs through passive diffusion [28,33]. Absorbed thiamine is converted, in the presence of magnesium, to thiamine pyrophosphate [34]. In healthy individuals, dietary thiamine absorption is greater than 95% of the vitamin B1 intake from food, provided there are no factors limiting its absorption. For example, products containing polyhydroxyphenols (e.g., coffee, tea) may reduce thiamine bioavailability by forming complexes or modifying the pH of gastric juice. Some foods (e.g., raw fish or tea) may contain thiaminases that degrade vitamin B1 [24,35]. The recommended daily intake of vitamin B1 for adults depends on gender and physiological condition and is 1.2 mg (men), 1.1 mg (non-pregnant and non-lactating women), or 1.4 mg (pregnant or breastfeeding women) [36].

Thiamine pyrophosphate constitutes 70–90% of the total endogenous vitamin B1. The distribution of thiamine and the proportion of individual phosphate forms in the tissues and fluid spaces of the human body is not uniform. In the human body, thiamine concentrations are generally highest in skeletal muscle, heart, brain, liver, and kidneys. Approximately 80% of thiamine in the blood is located in erythrocytes and approximately 15% in leukocytes, mainly in the form of TDP and TTP. In blood plasma, vitamin B1 occurs primarily in the free form or bound to albumin as TMP or TDP [24,25,35].

Vitamin B1 is excreted by the kidneys as inactive metabolites. In cases of excess intake, it is eliminated unchanged in the urine [25].

## 4. Vitamin B1 Deficiency—Causes and Clinical Effects

Thiamine deficiency may result from insufficient intake (deficiency diet, e.g., based on large amounts of refined rice or cassava roots), absorption disorders (e.g., gastric bypass, malabsorption syndrome), excessive loss (e.g., intense vomiting, chronic diarrhea, diuretics), increased demand (e.g., neoplastic growth, pregnancy, lactation, sepsis) or excessive consumption of ethyl alcohol (chronic alcoholism) [14,37]. Since thiamine has a short half-life (1–12 h) and the body can store it for 1 to 3 weeks, vitamin B1 deficiency develops after about 3 weeks of complete thiamine deficiency in the diet, which results in a significant reduction or complete depletion of its tissue reserves [28].

Thiamine deficiency results in metabolic disorders leading to metabolic acidosis with a significant increase in lactate and pyruvic acid levels. The nervous and muscular systems are most sensitive to the toxic effects of pyruvic acid. Symptoms of neuromuscular dysfunction include muscle cramps and pain, impaired memory, concentration, and cognitive abilities, nystagmus, chronic fatigue, and depressive symptoms. Swelling of the limbs, decreased libido, nausea, loss of appetite, and constipation, resulting in excessive weight loss, can also occur. Cardiac arrhythmias, including tachycardia, can occur [37,38,39,40]. Extreme vitamin B1 deficiency results in beri-beri disease or Wernicke–Korsakoff syndrome [26,39,41].

### 4.1. Beri-Beri Disease

Beri-beri primarily causes symptoms affecting the nervous system and circulatory system and can be severe. There are two forms of beri-beri (dry and wet) in adults, and an infantile form. Dry beri-beri (also known as neurological beri-beri) includes muscle weakness, numbness, and tingling, while wet beri-beri (also known as cardiovascular beri-beri) includes swelling, shortness of breath, and a rapid heart rate. Beri-beri in infants primarily manifests as loss of appetite, vomiting, lactic acidosis, changes in heart rate, and an enlarged heart. Infantile beri-beri develops in infants fed breast milk by mothers deficient in vitamin B1 [21,37].

### 4.2. Wernicke–Korsakoff Syndrome

Wernicke–Korsakoff syndrome is a neurodegenerative disease most commonly found in individuals with chronic alcoholism. It comprises two groups of sequential disorders: Wernicke’s encephalopathy (ataxia, ophthalmoplegia, and altered consciousness) and Korsakoff’s psychosis (memory disturbances, disorientation, confabulation, apathy, affective disturbances, and changes in emotions and social skills) [42,43,44].

### 4.3. Vitamin B1 Supplementation

Thiamine supplementation (thiamine hydrochloride) is recommended for therapeutic purposes (symptomatic deficiencies), prophylactically (increased risk of deficiency and/or increased thiamine demand), and as a supportive therapy, for example, in the treatment of rare congenital metabolic disorders, heart failure of various etiologies, herpes zoster, gestosis during pregnancy, and biliary and liver diseases. Therapeutic vitamin B1 can be administered via various routes: enterally or parenterally or transdermally. Parenteral administration is the route of choice primarily in situations where enteral administration would be ineffective, such as conditions of impaired absorption or excessive loss (nausea, vomiting, diarrhea, metabolic disorders, gastrointestinal surgery), and in situations requiring rapid replenishment of thiamine deficiency [45,46,47].

Vitamin B1 is considered a non-toxic substance, and thiamine supplementation is usually well tolerated and not associated with significant risk to the patient. Thiamine overdose administered enterally is unlikely because intestinal absorption of vitamin B1 at doses above 5 mg is inhibited, and with intramuscular administration, excess thiamine is excreted unchanged in the urine. The most commonly reported adverse effects associated with thiamine supplementation include nausea, urticaria, lethargy, ataxia, intestinal motility disorders, and minor injection site reactions. Anaphylactic shock (so-called thiamine shock) is very rare and generally occurs only after intravenous administration; therefore, intravenous administration of vitamin B1 is not recommended unless this form is necessary [25,33,48,49,50].

## 5. Vitamin B1 Hypersensitivity

The first brief clinical reports of possible hypersensitivity reactions to thiamine were published at the turn of the 1940s and 1950s [51,52,53,54,55,56,57,58,59,60,61]. According to Zaikov et al. [62], B vitamins cause approximately 4–5% of all reported drug hypersensitivity reactions, and of all B vitamins, thiamine is the most common allergen [63]. Based on retrospective analyses of data from various centers [48,49,50,64], the frequency of adverse reactions associated with vitamin B1 supplementation was estimated to be 0.86–1.1%. These reactions were typically associated with the administration of high doses of thiamine hydrochloride (100–500 mg) over a short period (intravenous bolus). The severity, clinical symptomatology, and intensity of the reported symptoms varied considerably. The majority (approximately 1.02%) were local reactions involving the skin (irritation, swelling, redness, and itching at the injection site). Generalized reactions occurred sporadically (0.093%) and were quite mild (generalized urticaria with pruritus or generalized edema) [48,49,50,64].

### 5.1. Vitamin B1 Hypersensitivity in Clinical Cases

Based on the available literature (Table 2), it can be noted that hypersensitivity to thiamine, including anaphylactic shock (very rare), may occur primarily after parenteral exposure to vitamin B1, such as intramuscular injection or intravenous infusion. Other routes of exposure to vitamin B1 (percutaneous, intradermal, airborne, or oral) very rarely cause hypersensitivity reactions, which in these situations are more likely to be local (limited to the site of exposure) or mild systemic (following oral exposure). It is worth noting that vitamin B1 may cause occupational allergy.

### 5.2. Vitamin B1 Hypersensitivity-Probable Etiopathogenetic Mechanisms

**Figure 3:**
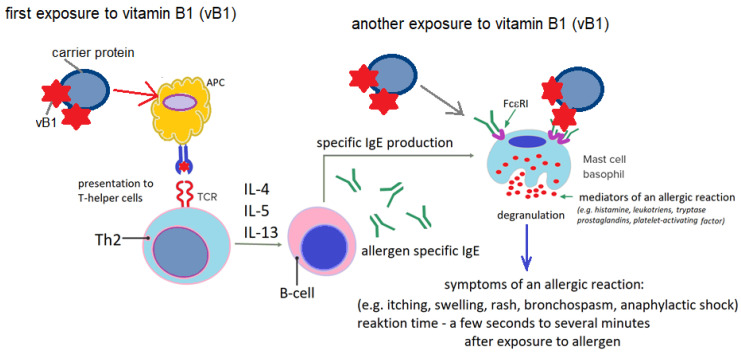
Hypersensitivity to thiamine in the immediate reaction mechanism (IgE-dependent); IL—interleukins; IgE—immunoglobulin E; Th2—T helper lymphocyte type 2; B-cell—B lymphocytes; APC—antigen-presenting cell; FcεRI—high-affinity receptor for IgE type I; vB1—vitamin B; author’s own figure.

The mechanism of hypersensitivity reactions to thiamine is not fully understood, but the clinical picture of the described events seems to indicate immediate-type hypersensitivity, dependent on immunoglobulin E (IgE). According to some researchers, it is likely that parenteral administration of thiamine causes vitamin B1, which has not been metabolized, to act as a hapten. As a hapten, thiamine binds to endogenous proteins, transforming it into a complete antigen (immunogen) [52,66] (Figure 3).

Based on the literature data (Table 2), it can be observed that oral thiamine is unlikely to be a sensitizing factor. This is likely due to the fact that vitamin B1 administered enterally is subject to metabolic changes. Vitamin B1 metabolites are released into the blood slowly, causing their concentration to increase gradually. It is likely that either the concentration of thiamine metabolites in the circulation does not reach the threshold values necessary to induce a hypersensitivity reaction, or the phosphorylation of thiamine, occurring during its metabolic transformation during resorption in the intestinal mucosa, deprives it of its hapten properties [75,80]. This conclusion is also supported by the observation that patients who experience a hypersensitivity reaction to thiamine after intravenous and/or intramuscular administration can take vitamin B1 orally without any adverse reactions [67,73,75].

The IgE-dependent nature of the reaction is also supported by the fact that the presence of thiamine-specific IgE antibodies has been detected in the blood of patients with hypersensitivity reactions to vitamin B1 [66,75,81]. It has also been observed that the serum concentration of thiamine-specific IgE antibodies gradually decreases until they disappear completely after approximately 11–18 months following an anaphylactic episode induced by vitamin B1. Therefore, it seems that the IgE-dependent nature of thiamine hypersensitivity is very likely, and the amino group of thiamine is responsible for the binding of IgE antibodies [52,66,75,81].

**Figure 4:**
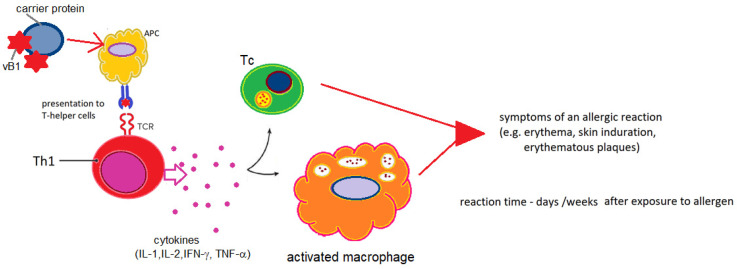
Hypersensitivity to thiamine in the mechanism of local/contact reaction (IgE-independent); IL—interleukins; TNF-α—tumor necrosis factor alpha; IFN-γ—interferon gamma; Th1—T helper lymphocyte type 1; Tc—cytotoxic lymphocyte; APC—antigen-presenting cell; vB1—vitamin B1; author’s own figure.

The variety of clinical manifestations of thiamine-induced hypersensitivity reactions and their dynamics mean that other mechanisms of sensitization to vitamin B1 cannot be ruled out, particularly when administered via routes other than intravenous or intramuscular injection. It is noteworthy that exposure through the skin [57,61,77,79] or via the airborne route [78,79] does not induce immediate systemic reactions but rather local reactions limited to the site of exposure, which often develop with a delay or after a longer period of exposure. This nature of the reaction seems to support a mechanism of delayed hypersensitivity independent of antibodies (Figure 4).

Other etiopathogenetic mechanisms that develop a hypersensitivity reaction to vitamin B1, or mixed mechanisms, cannot be ruled out. For example, Proebstle et al. [75] demonstrated, in addition to IgE, the presence of thiamine-specific IgG antibodies in the blood of a patient who developed a limited hypersensitivity reaction thirty minutes after injection of a substance containing vitamin B1 into the epicondyle. The patient’s concentrations of both classes of antibodies were monitored for 18 months after this event and it was noted that the decrease in specific IgE concentration was accompanied by a less dynamic but significant increase in thiamine-specific IgG concentration [75].

Due to the limited data available on the phenomenon of thiamine hypersensitivity, it is difficult to clearly determine the mechanism of these reactions. However, it seems likely that more than one classic etiopathogenic mechanism of hypersensitivity may be involved. The involvement of reactions occurring through the activation of the complement system, direct activation of mast cells, pseudoallergic reactions or allergy to additional substances (e.g., solvents or preservatives) contained in commercial pharmaceutical preparations of thiamine cannot be excluded. Unfortunately, the small number of reported events does not allow for a definitive resolution of this problem.

### 5.3. Vitamin B1 Hypersensitivity-Diagnostic Possibilities

Diagnosing thiamine hypersensitivity is associated with certain difficulties, which result, among other things, from the fact that vitamin B1 rarely causes such reactions, which means that it is not considered as a potential causative factor in the initial stages of diagnosis. As evidenced by the clinical data presented in Table 2, diagnosing vitamin B1 hypersensitivity based on standard (validated) diagnostic tests is currently not possible. The procedure relies primarily on a thorough clinical interview and a detailed patient history. Many authors have primarily used skin prick tests, intradermal tests, or epidermal patch tests with substances (drugs, cosmetics, etc.) indicated or provided by the patient (as is) and with individual ingredients of these preparations (in chemically pure form or coming from manufacturers of drugs or cosmetics that were the probable cause of hypersensitivity). Tests detecting specific IgE antibodies were unvalidated proprietary tests, whose use is for scientific and cognitive purposes only and has no application in routine clinical diagnosis. The lack of standardized controls, reference values and unified procedures prevents the use of this type of laboratory tests for diagnostic purposes.

The basophil activation test (BAT) appears to be a promising laboratory technique in the diagnosis of thiamine hypersensitivity. BAT has been successfully used in the diagnosis of hypersensitivity to various allergens, both proteinaceous and nonproteinaceous, including various food additives, drugs, and pharmaceuticals [82,83,84]. This procedure was successfully used by Gouveia et al. [85] in the diagnosis of folic acid hypersensitivity and by Morales-Hidalgo et al. [86] in the diagnosis of vitamin B12 hypersensitivity. The use of BAT in the routine diagnosis of thiamine hypersensitivity is currently limited by the lack of a standardized vitamin B1 solution, a unified analytical procedure, and defined cut-off values.

### 5.4. Vitamin B1 Desensitization as a Therapeutic Option for Thiamine Hypersensitivity

Thiamine hypersensitivity is a significant clinical problem in patients requiring vitamin B1 supplementation or in patients with clinical conditions requiring ongoing vitamin B1 supplementation. Due to the lack of alternative therapy, it is necessary to employ therapeutic strategies that lead to thiamine tolerance, which can be achieved through vitamin B1 desensitization [52].

**Figure 5:**
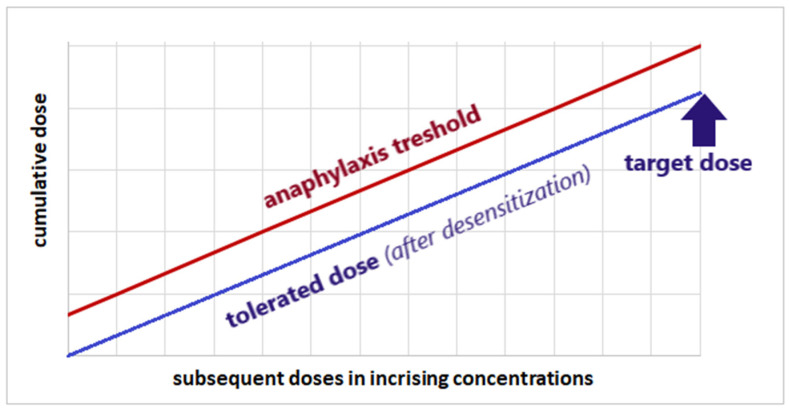
General assumptions (target and effects) of drug desensitization protocols—author’s own engraving [87,88,89,90,91,93].

Drug desensitization is used in patients requiring therapy with a specific drug to which they have experienced a hypersensitivity reaction and who have no alternative therapy available. The goal of this procedure is to induce tolerance to a specific drug, which is a prerequisite for continuing therapy with that pharmaceutical [87,88,89,90,91]. The desensitization protocol typically involves gradually increasing the drug dose, ultimately leading to a therapeutic dose. This strategy leads to a systematic increase in the threshold concentration, which inevitably leads to anaphylaxis. The mechanism of desensitization is based on the inhibition of effector cells (mast cells, basophils). This process occurs gradually thanks to the increasing concentration of each successive drug dose administered. Gradual inhibition of effector cells increases the threshold drug concentration necessary to induce clinical symptoms of a hypersensitivity reaction [87,88,89,92]. The initial dose of desensitization (initial dose) is usually 10 to 10,000 times lower than the target (therapeutic) dose. Desensitization can be performed in various schedules, with dose escalation occurring systematically at regular intervals. The interval between subsequent doses depends on the type of drug (mainly its pharmacokinetics) and the escalation of the patient’s symptoms. The route of drug administration in desensitization usually depends on the standard therapeutic route. Oral, sublingual, intramuscular, subcutaneous, or intravenous infusion (with gradually increasing flow) are used. The drug is administered until the intended target dose is reached [87,88,89,93] (Figure 5).

**Figure 6:**
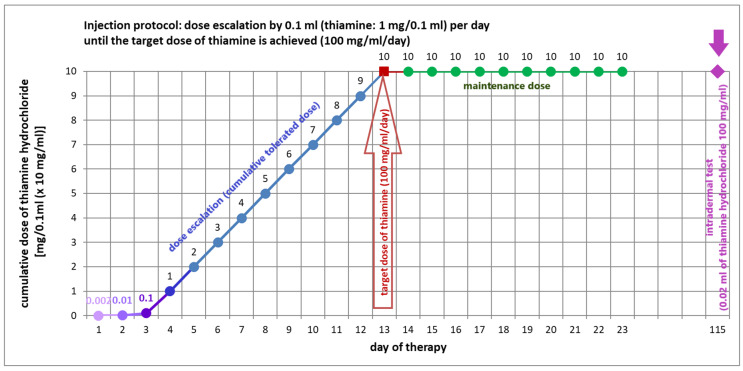
Scheme of desensitization with thiamine hydrochloride according to Mitrani [52]—author’s own engraving.

The only vitamin B1 desensitization protocol reported in the scientific literature was proposed in 1944 by Mitrani [52]. Desensitization was performed on a 15-year-old girl who developed hypersensitivity to thiamine administered by intramuscular injection (dose 50 mg/day) in the form of a macular rash on the face, chest, and back, associated with itching. The desensitization protocol (Figure 6) used a solution of thiamine hydrochloride in physiological saline (base concentration 100 mg/mL; 10 mg/0.1 mL). This solution was diluted with physiological saline in the following proportions: 1:5000, 1:1000, 1:100, and 1:10.

Desensitization was performed via intramuscular injection. The highest dilution (i.e., 0.002 mg thiamine/0.1 mL) was initiated. Subsequent doses (i.e., 0.01 mg thiamine/0.1 mL; 0.1 mg thiamine/0.1 mL; and 1 mg thiamine/0.1 mL) were administered in ascending doses, one per day. After achieving tolerance to the 1 mg thiamine/0.1 mL dose, the patient was administered daily in increments of 1 mg thiamine/0.1 mL until the target dose (i.e., 10 mg thiamine/0.1 mL; 100 mg thiamine/1 mL) was reached. The target dose was used as a maintenance dose, which was then administered daily for the next 10 days. The patient did not experience any hypersensitivity symptoms throughout the desensitization process [52]. Three months after the last maintenance dose, the patient underwent intradermal testing with 0.02 mL of thiamine hydrochloride (at a concentration of 100 mg/mL) against physiological saline (negative control) with negative results, confirming the efficacy of desensitization [52].

Thiamine desensitization appears to be an attractive and effective therapeutic option for individuals allergic to vitamin B1 who require vitamin B1 supplementation. This appears to be the optimal solution, especially if thiamine must be administered intravenously or intramuscularly and alternative therapeutic options are not available.

## 6. Summary and Conclusions

Thiamine is a water-soluble vitamin from the B vitamin group. It is widely present in many food products, particularly those of plant origin [24,29,30]. Under physiological conditions, the daily requirement for vitamin B1 is fully met by a properly balanced diet [20,21,24,28]. Certain clinical situations, such as dietary errors, increased demand, malabsorption, or excessive loss, may result in vitamin B1 deficiency [14,28,37]. Thiamine deficiencies can result in dysfunction of many organ systems, including significant dysfunction of the nervous system [37,38,39,40]. Such clinical situations require the implementation of vitamin B1 supplementation to replenish deficiencies and restore normal body function. Vitamin B1 can be administered by various routes (enterally and parenterally, including intramuscularly and intravenously). The dose and route of administration depend on the individual clinical condition of the patient [45,46,47].

It is estimated that 4–5% of patient-reported hypersensitivity reactions to drugs and other pharmaceuticals are caused by B vitamins [58]. According to Ou et al. [63], thiamine is the most common allergen of all B vitamins. However, an analysis of retrospective data from various centers [48,49,50,64] indicates that various types of hypersensitivity reactions associated with vitamin B1 administration occurred in less than 2% of all patients supplemented with thiamine, which allows us to assess this phenomenon as rather rare. Moreover, these reactions were usually mild and were most often associated with intravenous infusion of large doses of vitamin B1 over a short period of time. Oral administration of thiamine generally did not cause adverse reactions [48,49,50,64]. No cases of hypersensitivity to vitamin B1 related to the consumption of foods naturally containing thiamine or foods fortified with vitamin B1 have been reported (Table 2).

It is assumed that vitamin B1 is a hapten [52,66] and hypersensitivity reactions to thiamine probably occur via an immediate IgE-dependent mechanism [75,81], though, due to insufficient data, this cannot be unequivocally confirmed and mechanisms of hypersensitivity reactions, including late (cellular) reactions, can be excluded, especially in the case of local exposure to thiamine (skin, mucous membranes) [57,61,75,77,78,79].

Diagnostic procedures for suspected thiamine hypersensitivity are based primarily on a detailed clinical interview and analysis of the patient’s medical history, as well as skin prick, intradermal, and epidermal tests with the patient’s recommended preparations or their components. Standardized diagnostic tests are not available (see Table 2).

Patients who experience hypersensitivity reactions following parenteral administration usually tolerate oral thiamine (Table 2). Therefore, one therapeutic option for vitamin B1 hypersensitivity is to switch the supplementation form, provided there are no contraindications and that changing the administration form does not reduce the effectiveness of the therapy. Another therapeutic option is thiamine desensitization [51]. Vitamin B1 desensitization appears to be a particularly effective solution in situations where parenteral thiamine supplementation is necessary and changing the route of administration is not possible.

## Figures and Tables

**Table 1:** Vitamin B1 content in selected food products [24,29,30].

Plant Products	B1 mg/100 g	Animal Products	B1 mg/100 g	Other Products	B1 mg/100 g
Oranges	0.1	Pork tenderloin	1.1	Brewer’s yeast (dried)	15–20
Pineapple	0.08	Pork	0.54	Baker’s yeast (dried)	2.7–6.6
Banana	0.05	Pork liver	0.43		
Apple	0.04–0.12	Beef	0.7		
Blueberries	0.03	Beef liver	0.3		
Strawberries	0.03	Veal (heart)	0.6		
Peaches	0.03	Veal	0.09		
Whole wheat flour	0.55	Poultry (duck/goose)	0.1		
Whole rye flour	0.3	Salmon	0.17		
Brown rice	0.29	Trout	0.09		
Soybeans	0.85	Eggs	0.12		
White beans	0.6	Cheese	0.02–0.06		
Green peas	0.32	Cow’s milk	0.04		
Cauliflower	0.11	Breast milk	0.01		
Brussels sprouts	0.1				
Cabbage	0.05				
Potatoes	0.07				
Carrots	0.06				
Tomatoes	0.06				
Oranges	0.1				

**Table 2:** Hypersensitivity to vitamin B1 in clinical cases.

Author(s) (Year of Publication) [Reference Number]	Patient: Gender/Age	Clinical Symptoms	Product/Route of Administration	Diagnostics
Intravenous injection				
Reingold, I.M.; Web, F.R./1946 [54]	M/-	Fatal anaphylaxis	Thiamine hydrochloride/intravenously	Not done
Armanino, L.P.; Scott, W.S. (1950) [56]	M/39 y.	Wheezed and noted some pruritus shortly after the second injection Anaphylactic shock two or three minutes after another injection (4 months after the first episode)	Thiamine hydrochloride (10 mg)/intravenously	Not done
Hiratani, M. et al. (1992) [65]	F/54 y.	Anaphylactic shock	Vitamedin^®^ (a vitamin B complex; includes TDSP, 107.13 mg/vial; pyridoxine hydrochloride, 100 mg/vial; cyanocobalamin, 1 mg/vial; D-mannitol, 400 mg/vial)/intravenously	Intradermal test with TDSP-positive result. Patch test with TDSP with negative result
Fernandez, M. et al. (1997) [66]	F/52 y.	Systemic pruritus, generalized erythema, and hypotension (20 min after injection)	Thiamine hydrochloride in a combination drug (vitamins B1 and B12, Xylocaine, and dexamethasone)/intravenously	Intradermal test with single ingredients, with positive result for vitamin B1. Thiamine-specific IgE (ELISA)
Johri, S. et al. (2000) [67]	F/51 y.	Deep cyanosis, shallow, labored breathing, rhythm and blood pressure disorders (20 min after injection); three-time episode	Thiamine hydrochloride (100 mg)/intravenously	Not done
Takahashi, Y. et al. (2012) [68]	M/56 y.	Anaphylactic shock	Nornicicamin^®^ (TDSP, Pyridoxine Hydrochloride, Hydroxocobalamin)/intravenously	Intradermal test with TDSP-positive result Patch test with TDSP-positive result
Juel, J. et al. (2013) [69]	M/44 y.	Cardiac arrest due to anaphylactic shock	Thiamine hydrochloride (300 mg)/intravenously	Not done
Amano, A. et al. (2022) [70]	M/80 y.	Anaphylaxis (with generalized pruritus, dyspnea, and decreased blood pressure) immediately after injection	Vitamedin^®^ (a vitamin B complex, which includes TDSP, 107.13 mg/vial; pyridoxine hydrochloride, 100 mg/vial; cyanocobalamin, 1 mg/vial; D-mannitol, 400 mg/vial)/intravenously	Skin prick test with Vitamedin^®^ (as is), with positive result (wheal after 15 min and anaphylactic shock after 30 min). Histamine release test with the following: - Vitamedin^®^ (as is) dose-dependent positive result - TDSP dose-dependent positive result - Vitamin B1 derivatives (thiamine disulfide, benfotiamine, cocarboxylase)–weakly positive dose-independent results - Thiamine hydrochloride, negative results - Pyridoxine hydrochloride, negative results - Cyanocobalamin, negative results - D-mannitol, negative results
Kumagai, J. et al. (2023) [71]	F/20 y.	Anaphylactic shock	multivitamin witch TDSP/intravenously	Intradermal test with the following: - 1% multivitamin–positive result - TDSP–positive result
Intramuscular injection				
Mitrani, M.M. (1944) [52]	F/15 y.	Maculo-pruriginous eruption on the face, chest, and on the back (where it was of great intensity and extent) after the first injection (the episode repeated three times after subsequent injections)	Thiamine hydrochloride (50 mg)/intramuscularly	Intradermal tests with thiamine hydrochloride, with positive result
Tetreault, A.F.; Beck, A.F. (1956) [60]	M/62 y.	Anaphylactic shock	Thiamine hydrochloride/intramuscularly	Not done
Van Haecke, P. et al. (1995) [72]	F/86 y.	Fatal anaphylaxis (2 h after injection)	Thiamine hydrochloride (250 mg)/intramuscularly	Not done
Aurich, S. et al. (2018) [73]	F/78 y.	Anaphylaxis after the fourth injection	Thiamine hydrochloride/intramuscularly	Skin prick test with pure commercially available aqueous preparations thiamine hydrochloride, with positive result. Single-blinded, placebo-controlled oral challenge test witch thiamine hydrochloride, and with negative result
Rodríguez-Fernández A. et al. (2018) [74]	F/50 y.	Generalized urticaria, edema and facial erythema after 30 min of intramuscular administration	Inzitan^®^ (cyanocobalamin, dexamethasone, pyridoxine, thiamine (50 mg; 25 mg/mL), lidocaine)/intramuscularly	Skin prick test with Inzitan^®^ (as is), with negative result. Intradermal test with Inzitan^®^ ingredients (singly), with thiamine-positive result and rest of ingredients, with negative result
Subcutaneous/intradermal injection				
Laws, C.L. (1941) [51]	F/72 y.	Redness and swelling of the eyes, ears, generalized hives, chest tightness 30 min after injection (during the next treatment series)	Thiamine hydrochloride/subcutaneously	Intradermal tests with the drug used (as is), with positive result
Proebstle, T.M. et al. (1995) [75]	M/47 y.	Itching of the hands, trunk and neck 30 min after injection. Anaphylaxis 5 min after the second injection (two weeks later)	Thiamine hydrochloride (in a combined drug)/paraepicondyle injection	Thiamine-specific IgE (ELISA) Thiamine-specific IgG (ELISA) Skin prick tests with thiamine hydrochloride, with positive result
Oral administration				
Osman, M.; Casey, P. (2013) [76]	F/47 y.	Swelling of both legs, slightly painful, feeling of heaviness in the legs (after 7 days of supplementation; after 4 days of discontinuing the supplementation the symptoms disappeared). Symptoms of similar dynamics and nature reappeared during the next thiamine supplementation 18 months later	Thiamine hydrochloride (200 mg/day)/orally	Not done
Transdermal administration				
Combes, F.C.; Groopman, J. (1950) [57]	F/35 y.	Dermatitis of the hands and forearms (area of direct exposure to vitamin B1)	Occupational exposure to liquid vitamin B1 (thiamine hydrochloride)	Patch testing with substances related to occupational exposure, with positive result for vitamin B1
Hjorth, N. (1958) [61]	F/17 y.	Hand dermatitis (after 4 months of exposure to thiamine) resolving due to the lack of exposure and recurring after re-exposure	Occupational exposure to liquid vitamin B1 (thiamine hydrochloride)	Patch tests with substances related to occupational exposure, with positive result for thiamine, cocarboxylase and 2-methyl-6-amino-5-brom-methyl-pyrimidine. Intracutaneous tests with thiamine-positive result
Arruti, N. et al. (2013) [77]	F/46 y.	Localized, limited, pruritic, micropapular erythematous rash	Voltaren^®^ (diclofenac) and Inzitan^®^ (lidocaine, dexamethasone, cyanocobalamin (vitamin B12) and thiamine (vitamin B1))/topical application by iontophoresis	Patch tests with: - the TRUE Test^®^ system (positive results with nickel and epoxy resin) - the implicated drugs (as is)–negative results Skin prick tests with the implicated drugs (as is), negative results. Intradermal tests with the implicated drugs (as is), negative results. Oral challenge test with diclofenac, negative results. Intramuscular challenge test with Inzitan^®^, positive results (24 h after injection, local reaction in the area of previous transdermal application). All tests repeated with each Inzitan^®^ component-positive reactions after thiamine challenge test
Airborne (inhalation) exposure				
Drought, V.J. et al. (2005) [78]	M/46 y.	Bronchial asthma	Occupational exposure to vitamin aerosol containing thiamin hydrochloride	spirometry, reduction of respiratory function parameters after occupational exposure to thiamine when compared with the test results on days free from exposure
	M/43 y.			
Transdermal administration and/or airborne (inhalation) exposure				
Ingemann, L.A. et al. (1989) [79]	M/54 y.	Recurrent itchy eczema on the forearms, hands and face (after a month of exposure)	Occupational exposure to vitamin B1 (thiamine hydrochloride) dust	Patch tests with thiamine (10%, 5%, 1% aq) and thiothiamine (5%, 1% aq), positive results Patch tests (European standard), negative results
	M/32 y.	Itchy eczema (localized oozing) on hands and feet that spread to the rest of the body (after one month of exposure)	Occupational exposure to vitamin B1 (thiamine hydrochloride) dust. Long-term oral supplementation with vitamin preparations (with vitamin B1)	Patch test with thiamine hydrochloride (10% aq)-Positive result Patch test (European standard), positive results for a mixture of formaldehyde and fragrances

Legend: y.—years; TDSP: Thiamine disulfide phosphate; ELISA: Enzyme linked immunosorbent assay; IgE: Immunoglobulin E; IgG: Immunoglobulin G.

## Data Availability

No new data were created or analyzed in this study.
